# Supplementary material for: Regulation of Fructose 1,6-Bisphosphatase in Procyclic Form Trypanosoma brucei
Source: Pathogens. 2021 May 18;10(5):617. doi: 10.3390/pathogens10050617 (PMC8157246; doi:10.3390/pathogens10050617)
Supplement: Supplementary file 1 [file pathogens-10-00617-s001.zip › pathogens-1204557-supplementary.pdf]

Supplemental Data

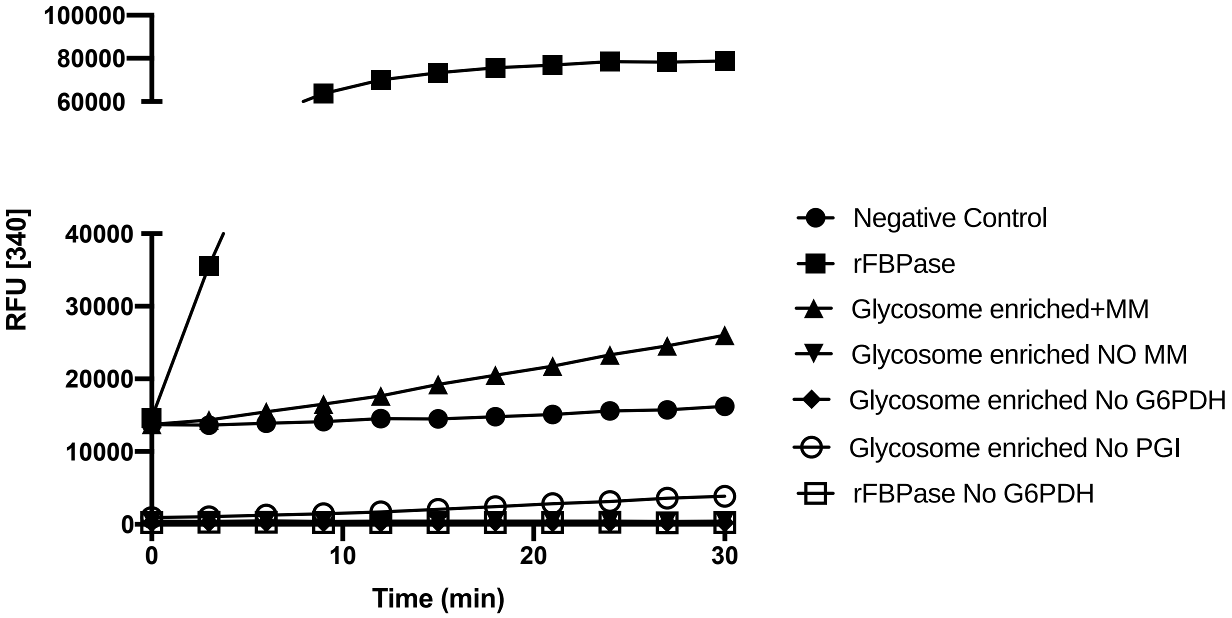

Figure S1: FBPase assays. RFU [340] was measured for reactions containing glycosome enriched fractions and master mix (MM) containing PGI and G6PDH (solid triangles), glycosome enriched fractions without MM (solid inverted triangles), glycosome enriched fractions and MM without G6PDH (solid diamonds), glycosome enriched fractions with MM without PGI (open circles) and rFBPase with MM without G6PDH. Negative control, master mix without glycosome enriched fractions (solid circles).
